# Autonomic dysfunction in reversible cerebral vasoconstriction syndromes

**DOI:** 10.1186/1129-2377-14-94

**Published:** 2013-11-25

**Authors:** Shih-Pin Chen, Albert C Yang, Jong-Ling Fuh, Shuu-Jiun Wang

**Affiliations:** 1 Faculty of Medicine, National Yang-Ming University School of Medicine, Taipei, Taiwan; 2 Department of Neurology, Neurological Institute, Taipei Veterans General Hospital, Taipei, Taiwan; 3 Department of Psychiatry, Taipei Veterans General Hospital, Taipei, Taiwan; 4 Institute of Brain Science, National Yang-Ming University, Taipei, Taiwan

**Keywords:** Reversible cerebral vasoconstriction syndromes, Thunderclap headache, Autonomic nervous system, Heart rate variability

## Abstract

**Background:**

Autonomic imbalance may play an important role in the pathogenesis of reversible cerebral vasoconstriction syndromes (RCVS). This study aimed to assess the autonomic function by analyzing heart rate variability (HRV) in patients with RCVS.

**Methods:**

Patients with RCVS and age- and gender-matched controls were consecutively recruited. All patients (both ictal and remission stage) and controls underwent 24-hour ambulatory electrocardiographic (ECG) recordings. HRV measures covering time and frequency domains were used to assess autonomic functioning.

**Results:**

Thirty-nine patients with RCVS and 39 controls completed the study. Compared to the controls, RCVS patients during the ictal stage showed reductions in parasympathetic-related indices, including the root mean square of difference of consecutive interbeat intervals (RMSSD) (22.1 ± 7.0 vs. 35.2 ± 14.2, p < 0.001), the percentage of adjacent intervals that varied by more than 50 ms (pNN50) (3.7 ± 3.4 vs. 10.6 ± 8.1, p < 0.001), and high-frequency power (HF) (5.82 ± 0.73 vs. 6.77 ± 0.74; p < 0.001), and increased low-frequency/high-frequency (LF/HF) ratio (index of sympathovagal balance) (3.38 ± 1.32 vs. 2.48 ± 1.07; p =0.001). These HRV indices improved partially but remained significantly different from controls during remission.

**Conclusions:**

Decreased parasympathetic modulations and accentuated sympathetic activity might be a biological trait in patients with RCVS.

## Background

Reversible cerebral vasoconstriction syndrome (RCVS) is a unifying term for a variety of clinical-radiological syndromes characterized by recurrent thunderclap headaches and reversible multifocal cerebral vasoconstrictions [1-4]. Many secondary causes have been identified [1,3,4]; however, in a substantial group of patients, RCVS occurs spontaneously without any precipitating factors [2,3]. RCVS is not uncommon and potentially devastating because of high risks of complications such as posterior reversible encephalopathy syndrome (PRES), ischemic stroke, intracerebral hemorrhage (ICH) or cortical subarachnoid hemorrhage (SAH) [3,5-9]. The severity of vasoconstrictions is associated with PRES or ischemic stroke [6,7].

Despite gradual delineation of the clinical presentation, the underlying pathophysiology of RCVS remains enigmatic. An aberrant central sympathetic response has been proposed to play an important role [10], which can be partly supported by the fact that RCVS can occur in some patients with pheochromocytoma [11] and after the use of sympathomimetics [12,13]. Besides, the observations of blood pressure surge and/or the Valsalva maneuver-like triggers with elevated sympathetic tone also heightened the role of autonomic dysregulation in the pathogenesis [2]. However, to our knowledge no study has directly investigated the roles of the autonomic system in the pathogenesis of RCVS.

Analysis of heart rate variability (HRV), an established method to study abnormalities of the autonomic function [14], is an appropriate and easily applicable method to validate these speculations. Therefore, the present study employed standardized HRV measures to investigate the autonomic function in patients with RCVS and attempted to provide plausible pathophysiological mechanisms for this unique disorder.

## Methods

### Study subjects

We recruited consecutive patients presenting with acute severe headaches from the headache clinic and emergency department at Taipei Veterans General Hospital (TVGH) from June 2006 to September 2010. Subjects were excluded if they 1) were younger than 18 or older than 65 years, 2) had severe cardiac arrhythmia, such as continuous bigeminy, atrial fibrillation or atrial flutter, 3) had diabetes mellitus, 4) had experienced a severe or acute medical illness within 3 months preceding the study, and 5) had been placed on medications with a well-documented effect on HRV, such as tricyclic antidepressants with anti-cholinergic effects or beta blockers, etc. [14]. Each subject completed a detailed headache intake form and provided comprehensive medical and headache histories before undergoing clinical and neurological examinations. Brain magnetic resonance imaging (MRI), MR venography and MR angiography were performed to exclude intracranial lesions attributable to the patients’ headache. Spinal tap with cerebrospinal fluid (CSF) analysis was performed to help diagnosis if patients agreed. Subjects were hospitalized to expedite completion of these diagnostic investigations if condition allowed. If cerebral vasoconstrictions were demonstrated on the initial MRA, sequential MRA was arranged to evaluate their reversibility. Transcranial color-coded Doppler sonography (TCCS) was performed on the same day of the corresponding MRA. The methodology and follow-up protocol of MRA and TCCS were detailed elsewhere [6]. The participants were instructed to keep a headache diary to optimize the diagnosis and treatment. The diagnosis of RCVS required fulfillment of the following criteria: (1) at least two acute-onset severe headache (thunderclap headache), with or without focal neurological deficits; (2) vasoconstrictions demonstrated on magnetic resonance angiography (MRA); (3) reversibility of vasoconstrictions, as demonstrated by at least one follow-up MRA within 3 months; and (4) SAH or other intracranial disorders ruled out by appropriate investigations, but cortical SAH in RCVS was allowed. The diagnostic criteria were based on the definition of “benign (or reversible) angiopathy of the central nervous system” proposed by the International Classification of Headache Disorders, second edition (ICHD-2) (Code 6.7.3) [15] and the essential diagnostic elements of RCVS proposed by Calabrese et al. [1]. In addition, age- and gender-matched controls were recruited from colleagues, their relatives, or a databank of controls of other autonomic studies. Control subjects were eligible only when they were devoid of any systemic diseases except for grade 1 hypertension (SBP < 160 mmHg and DBP < 100 mmHg) [16] and migraine. Hypertension and migraine were allowed because they were not uncommon in patients with RCVS. The study protocol was approved by the TVGH Institutional Review Board. All patients signed informed consent before entering the study.

### Continuous electrocardiographic (ECG) monitoring

Subjects during the ictal stage received 24-hour ambulatory ECG monitoring using a Holter monitor (MyECG E3-80 portable recorder, Microstar Inc., Taipei, Taiwan) upon entering the study. This ECG study was defined as *ictal ECG.* Headache abortive treatment with painkillers was allowed during the 24-hour ECG monitoring if the patients had headache attacks, but nimodipine treatment was not implemented since a diagnosis of RCVS had not been confirmed meanwhile. In order to prospectively monitor the change of autonomic function in these patients, patients were asked to receive a follow-up ECG monitoring when their RCVS remitted (i.e. vasoconstriction had normalized and the patients had discontinued nimodipine treatment for at least one month), which was defined as a *remission ECG*. For the controls, no follow-up ECG monitoring was implemented. The Holter device continuously recorded three channels of ECG signals at a sampling rate of 250 Hz. The digitalized ECG signals were then stored on the computer for subsequent off-line HRV analysis by the custom software according to the open source of HRV algorithms [17]. The HRV analysis was performed by the same investigator (A.C.Y.) who was blind to clinical data.

### Heart rate variability analysis

HRV measures covering time and frequency domains were used in the analysis of R-R interval time series according to international guidelines [14]. Time domain HRV measures were derived for 24-hour time period, including mean heart rate, the standard deviation of the normal interbeat intervals (SDNN), the root mean square of difference of consecutive interbeat intervals (RMSSD), and the percentage of adjacent intervals that varied by more than 50 ms (pNN50) [18]. The SDNN assesses the overall variability of interbeat intervals. The RMSSD and pNN50 measure the short-term variation of interbeat intervals which is mainly modulated by the parasympathetic activity [19].

Spectral HRV measures include high-frequency power (HF; 0.15–0.40 Hz), low-frequency power (LF; 0.04–0.15 Hz), very-low-frequency power (VLF; 0.003-0.04 Hz), and LF/HF ratio. These spectral HRV indexes were averaged from non-overlapping 30-minute segments for the entire 24-hour time period. The power spectra of each 30-minute segment of R-R interval were computed by fast-Fourier transformation, with application of a Hanning window to minimize spectral leakage. VLF power is correlated with long-term fluctuations of interbeat intervals and is believed to be mediated partly by the renin-angiotensin-aldosterone system [14]. LF power has been reported to be modulated by both sympathetic and parasympathetic activities, whereas the HF power is mainly modulated by parasympathetic activity [20-22]. The LF/HF ratio represents the balance of autonomic system and is thought to be an indicator of the sympathetic activity [14,23].

### Statistical analysis

Descriptive statistics were presented as means ± standard deviation or percentages and median with range for non-normal distribution data. The spectral HRV indices were log transformed to produce normalized distributions. Chi-square was used to compare the categorical variables. Student’s *t-*test or paired *t-*test was used to determine whether there were differences in HRV indices between patients during ictal stage, remission stage, and controls. For correlations between two continuous variables, Pearson correlation coefficient *r* was adopted. A *p* value of less than 0.05 (two-tailed) was required for statistical significance. Because these HRV variables were highly correlated (14), corrections for multiple comparisons were not applied. All analyses were performed with the IBM SPSS Statistics software package, version 18.0.

## Results

### Clinical characteristics

During the study period, we prospectively recruited 39 patients with RCVS who had both eligible ictal and remission ECG monitoring. Thirty-nine subjects without major medical and psychiatric disorders were recruited as controls. The comparisons of demographics and associated medical conditions between patients and controls are listed in Table 1. The ictal ECG monitoring was conducted at a median of 12.5 (range 3–45) days after headache onset. The remission ECG monitoring was performed at a median of 178 (range 43–247) days after headache remission, at a time point that the MRA vasoconstrictions of the participants had normalized for 107.5 (range 33–158) days.

**Table 1:** The comparison of demographics, menopausal status and current medical illness between patients with RCVS and controls

	**Patients with RCVS**	**Controls**	***p *** **value**
	**(N = 39)**	**(N = 39)**	
Mean age ± SD, years	49.5 ± 10.4	50.0 ± 4.7	0.56
Sex (M/F)	0/39	0/39	1.00
Hypertension, *n* (%)	3 (7.7)	3 (7.7)	1.00
Diabetes, *n* (%)	0 (0)	0 (0)	1.00
Migraine, *n* (%)	8 (20.5)	6 (15.3)	0.77
Menopause, *n* (%)	18 (46.2)	19 (48.7)	1.00

*RCVS*, Reversible cerebral vasoconstriction syndromes.

Headaches in 79% of the patients with RCVS were explosive at onset, 69% reported throbbing headache after onset. Fifty-six percent reported nausea as an accompanying symptom, followed by phonophobia (38%), photophobia (38%), and vomiting (36%). Triggers were noted in 79% of study patients with the leading ones being: Defecation (41%), exertion (31%), bathing (28%), sex (26%), cough (18%) and rage (10%). The headaches remitted at a median of 14 (range 5–45) days. Three patients developed PRES and one patient developed acute ischemic stroke.

### Parasympathetic-related HRV indices

The parasympathetic-related HRV indices including RMSSD, pNN50, and HF power were significantly lower in patients during the ictal stage compared with the controls (Table 2). These indices improved partially during the remission stage when compared with indexes during the ictal stage (paired *t*-test), but remained lower than those of controls (Table 2). In fact, an ascending trend was observed in patients during the ictal stage, followed by the remission stage and controls.

**Table 2:** Comparison of heart rate variability profiles between patients with RCVS in ictal and remission stages and controls

	**Controls**	**RCVS patients (n = 39)**				
	**(n = 39)**	**Ictal stage**	P value*	**Remission stage**	P value*	P value**
**Time domain variables**						
SDNN, ms	129.4 ± 34.7	109.9 ± 24.8	0.006	120.7 ± 24.9	0.207	0.041
RMSSD, ms	35.2 ± 14.2	22.1 ± 7.0	<0.001	26.7 ± 11.1	0.005	0.019
pNN 50,%	10.6 ± 8.1	3.7 ± 3.4	<0.001	5.6 ± 6.3	0.003	0.070
**Frequency domain variables**						
Total power, ln (ms^2^/Hz)	9.01 ± 0.50	8.47 ± 0.48	<0.001	8.71 ± 0.40	0.005	0.008
VLF (0.003 – 0.04 Hz), ln (ms^2^/Hz)	8.66 ± 0.48	8.21 ± 0.46	<0.001	8.43 ± 0.37	0.017	0.011
LF (0.04 – 0.15 Hz), ln (ms^2^/Hz)	7.32 ± 0.60	6.63 ± 0.62	<0.001	6.90 ± 0.53	0.001	0.020
HF (0.15 – 0.4 Hz), ln (ms^2^/Hz)	6.77 ± 0.74	5.82 ± 0.73	<0.001	6.21 ± 0.75	0.001	0.016
LF/HF, ratio	2.48 ± 1.07	3.38 ± 1.32	0.001	2.97 ± 1.25	0.067	0.013

Values presented are mean ± SD.

*Comparison between patients and controls using Student’s t-test. ** Comparison between ictal and remission stage using paired-t test.

*HF*, High frequency power; *LF*, low frequency power; *pNN50*, Percentage of difference of successive interbeat intervals which are larger than 50 ms; *RCVS*, Reversible cerebral vasoconstriction syndromes; *RMSSD*, The square root of the mean of the sum of the squares of differences between adjacent normal interbeat intervals; *SDNN*, Standard deviation of normal inter-beat intervals; *VLF*, Very low frequency power.

### Sympathetic-related HRV index

The LF/HF ratios were significantly higher in patients during the ictal stage compared with the controls based on 24-hour HRV measures (Table 2). The consistent finding was found when diurnal or nocturnal HRV was compared between controls and patients during the ictal stage. These higher LF/HF ratios partially normalized during the remission stage (a nominal difference was noted when compared with that of controls) (Table 2).

### Other HRV indices

As the data in Table 2 demonstrated, patients during the ictal stage had significant reductions in SDNN, the total power and VLF, which were partially reversed but not normalized when patients were in the remission stage.

### The correlations of clinical variables and HRV indices

Because of small case number of patients with PRES (n = 3) and ischemic stroke (n = 1) in our study participants, statistics were not conducted to correlate the HRV indices with these complications. Nevertheless, patients with PRES had nominally lower total power and VLF of HRV (total power: 8.04 ± 0.58 vs. 8.52 ± 0.47; VLF: 7.83 ± 0.55 vs. 8.26 ± 0.46), lower parasympathetic indices (RMSSD: 16.3 ± 6.4 vs. 22.5 ± 6.7; pNN50: 1.8 ± 2.0 vs. 3.82 ± 3.41; HF power: 5.21 ± 1.05 vs. 5.86 ± 0.70) and elevated LF/HF ratio (4.13 ± 1.54 vs. 3.36 ± 1.25) than those without PRES (n = 36). The other clinical variables such as blood pressure surge, types and numbers of triggers, headache duration, date of ECG performance from headache onset (the ictal stage and the remission stage were calculated separately), history of hypertension or migraine, or menopausal status did not correlate with any HRV indices.

## Discussion

The study showed that patients with RCVS had markedly decreased heart rate variability, attenuated parasympathetic modulations and heightened sympathetic activity during the ictal stage. These autonomic aberrancies improved partially after disease remission, but were still abnormal in comparison with that of controls. These findings suggested that the autonomic dysfunction is not simply an accompanying phenomenon during the diseased stage but a biological trait marker in patients with RCVS.

The proposed pathophysiology of RCVS has been linked to sympathetic overactivity based on clinical observations or hypotheses including central vascular tone changes [8,24], aberrant sympathetic response [10], blood pressure surge [5], triggers [3,5], sympathomimetic agents [3,12,13], pheochromocytoma and hypertensive crises [25], and autonomic dysreflexia [26], etc. Our findings provided more direct evidence in support of these speculations. A remarkably decreased parasympathetic modulation was unexpected from clinical observations, but not irrational. Actually, the balance between the sympathetic and parasympathetic systems is like the balance of yin and yang [27]. For example, one arm weakened could actually make the other arm unantagonized and prone to a diseased state. It is thus possible that a dysfunctional autonomic nervous system renders the individual more susceptible to thunderclap headaches by exaggeratedly reacting to certain triggers, especially Valsalva maneuver-like triggers, and at a lower threshold. Cerebral blood vessels which were richly innervated by sympathetic nerve fibers [28,29], might also react to the heightened and unantagonized sympathetic activities and exhibit features of multifocal vasoconstrictions. Although it is likely that the pathophysiology of RCVS is multifactorial, we believe that central autonomic dysregulation plays a crucial role based on the distinct clinical presentations and the findings of this study.

It has been observed that during sympathetic activation the resulting tachycardia is usually accompanied by a marked reduction in total power, which may influence the change in HF and LF power in the same direction and thus lead to a less prominent ratio of LF over HF [14,30]. Hence, the higher LF/HF ratio observed in patients was still underestimated by the presence of decreased total power of HRV. Such sympathetic overactivity exactly coincided with the previously proposed neurogenic mechanism [10] and the foregoing deduction. Admittedly, one could speculate that an alternative explanation may be that the sympathetic overactivity was the consequence rather than the cause of severe headaches since similar trends have been observed in certain painful conditions [31-35]. However, such autonomic dysfunction should normalize after removal of the inciting factors. The persistently abnormal autonomic modulation during the remission stage effectively argues against this speculation.

Because all the enrolled patients had spontaneous RCVS, this study avoided the influence of HRV from exogenous factors, such as sympathomimetic agents, thus allowing us to examine the genuine endogenous autonomic derangements in these patients. It is noteworthy that low HRV measures are associated with onset and poor prognosis of cardiovascular diseases [36,37]. Since the case number of patients exhibiting PRES or ischemic stroke is limited, we were unable to accredit the prognostic value of HRV in predicting complications in patients with RCVS although a trend of lower HRV in found in patients with complications. However, given that the extent of lower HRV was not as severe as those with cardiovascular diseases, the prognosis of RCVS might not be as grave using similar criteria [36,37].

Our study had limitations. First, naturally one cannot study RCVS before it happens so that whether the abnormal cardiovascular autonomic responses in the RCVS group are the result of the event or causal to it could not be answered by the data. Second, HRV is highly variable among individuals and can be affected by confounders such as age, gender, medical conditions including hypertension [38] and migraine [35], as well as menopausal status [39], etc. However, our study design had attempted to exclude possible confounders by enrolling matched controls and excluding subjects with extreme ages and secondary RCVS. One study had shown that migraineurs with disabling attacks may be prone to hypofunction of autonomic nervous system, but the causality between the autonomic hypofunction and migraine was unknown [40]. It is possible that autonomic dysfunction is a common finding in patients with migraine or other severe headaches; however, the difference in study design and employed parameters made it difficult to directly compare our findings with those of previous studies. Whether there is difference in the severity of autonomic dysfunction between RCVS and migraine or other severe headaches requires further sophisticatedly designed studies which also take disease course into accounts. Of note, RCVS is known as a monophasic disorder; whereas, it might be difficult to define a true remission stage and the effects of therapeutics of primary headache disorders, such as migraine. Third, we are uncertain whether the use of nimodipine in our patients might have contributed to the changes in HRV during follow-up since calcium channel blockers had been reported to affect HRV [41,42]. To reduce the interference from this effect, we structured this study to allow a wash-out period from the use of the nimodipine. Finally, the nature of our study method reflected the autonomic controls on heart rates instead of directly on the control of cerebral vascular tone. More exploratory studies are required to investigate the relationship between central autonomic regulation and cerebral vasoconstrictions.

## Conclusion

Our study suggests that patients with RCVS have markedly deranged autonomic function, namely decreased heart rate variability, attenuated parasympathetic modulations and heightened sympathetic activity in the ictal stage. The autonomic derangement improved partially during the remission stage but still deviated from those of controls. The autonomic dysfunction might be a trait rather than state in patients with RCVS, which makes them more vulnerable to external triggers.

## Abbreviations

CSF: Cerebrospinal fluid; ECG: Electrocardiographic; HF: High-frequency power; HRV: Heart rate variability; ICH: Intracerebral hemorrhage; LF: Low-frequency power; MRI: Magnetic resonance imaging; pNN50: The percentage of adjacent intervals that varied by more than 50 ms; PRES: Posterior reversible encephalopathy syndrome; RCVS: Reversible cerebral vasoconstriction syndromes; RMSSD: The root mean square of difference of consecutive interbeat intervals; SAH: Subarachnoid hemorrhage; SDNN: Standard deviation of the normal interbeat intervals; TCCS: Transcranial color-coded Doppler sonography; VLF: Very-low-frequency power.

## Competing interests

The authors declare that they have no competing interests.

## Authors’ contributions

SPC participated in study design, acquisition of data, analysis and interpretation, as well as manuscript writing. ACY carried out data aquisition, analysis, interpretation, and manuscript writing. JLF participated in study design, study supervision, and critical revision of the manuscript for important intellectual content. SJW conceived of the study, participated in its design, coordination, supervision, and helped to draft and revise the manuscript. All authors read and approved the final manuscript.
